# Gastric antral vascular ectasia in a patient with lupus undergoing hemodialysis: a case report

**DOI:** 10.1186/s12882-020-02140-w

**Published:** 2020-11-10

**Authors:** Seok Hui Kang, A Young Kim, Jun Young Do

**Affiliations:** Department of Internal Medicine, Yeungnam University Hospital, 317-1 Daemyung-Dong, Nam-Ku, Daegu, 705-717 South Korea

**Keywords:** Gastric antral vascular ectasia, Lupus erythematosus, Hemodialysis, Anemia, Case report

## Abstract

**Background:**

Gastric antral vascular ectasia (GAVE), associated with autoimmune diseases, such as systemic lupus erythematosus, and hepatic or renal disorders, is a rare cause of gastrointestinal bleeding. We report the case of a patient with lupus erythematosus undergoing hemodialysis with an uncorrectable anemia caused by GAVE.

**Case presentation:**

A 76-year-old Korean woman with lupus undergoing hemodialysis frequently complained of symptoms or signs associated with anemia, such as dizziness, dyspnea, hypotension, melena, and hematemesis. Gastrointerstinal endoscopy revealed multiple erythematous and hyperemic mucosal lesions at the distal antrum without active bleeding, a finding compatible with GAVE. Although she frequently complained of symptoms or signs associated with anemia and had frequent gastrointestinal endoscopies with or without pre-emptive argon plasma coagulation, her clinical status is relatively stable, and she is undergoing maintenance hemodialysis without anticoagulants.

**Conclusion:**

This clinical case suggests that GAVE should be considered as a cause of the anemia resistant to erythropoiesis-stimulating agents and iron supplementation in patients with chronic kidney disease and lupus.

## Background

Anemia is a common complication in patients on hemodialysis (HD) [1]. Almost all patients undergoing maintenance HD have anemia, mainly due to decreased erythropoietin production or a functional or absolute iron deficiency. Other causes of anemia include infections, underdialysis, hyperparathyroidism, and malignancy. Gastrointestinal bleeding can also cause an uncorrectable anemia despite proper supplementation of erythropoietin and iron.

Gastric antral vascular ectasia (GAVE), associated with autoimmune diseases, such as systemic lupus erythematosus (SLE), and hepatic or renal disorders, is a rare cause of gastrointestinal bleeding [2]. Although the pathogenesis of GAVE is not fully understood, it can be an important cause of severe anemia in patients on HD. We report the case of a patient with SLE undergoing HD with an uncorrectable anemia caused by GAVE.

## Case presentation

A 76-year-old Korean woman was admitted to our hospital due to sore throat and poor oral intake. The patient presented with a decreased urine output and a febrile sensation. She had hypertension for 1 year and was taking amlodipine 10 mg/day. She had no history of gastrointestinal, liver, or renal diseases. In addition, she had undergone upper and lower gastrointestinal endoscopies (GFS), which revealed non-significant findings without bleeding lesion.

On admission, the blood pressure was 130/70 mmHg, with a pulse rate of 80 beats/min. Grade 1 pretibial pitting edema was noted. The laboratory findings were as follows: hemoglobin 8.6 g/dL (normal: 12–16 g/dL), platelet count 86,000/mm^3^, blood urea nitrogen 124.7 mg/dL (normal: 10 ~ 20 mg/dL), and serum creatinine 8.87 mg/dL (normal: 0.6 ~ 1.2 mg/dL). The 24-h urine protein was 1214 mg/day (urine volume was 1250 cc). Physical examination revealed a dehydrated tongue and no organomegaly. The rectal examination was negative for melena. Chest radiography and computed tomography showed ground-glass opacity in both lung fields and bilateral pleural effusion. Echocardiography showed a pericardial effusion without regional wall motion abnormalities. Abdominal computed tomography showed non-specific findings; the right and left kidneys were 8.6 and 8.9 cm in diameter, respectively. Antinuclear antibody (ANA) titer was 1:320. The patient had normal complement level and negative result for anti-dsDNA level. The diagnosis of SLE was performed using 2019 European League Against Rheumatism/American College of Rheumatology classification criteria [3]. The patients had high ANA level (1:320 titre) and SLE was considered. We evaluated additive criteria and the patient had thrombocytopenia (86,000/mm^3^, 4 score weightage), pleural and pericardial effusion (detected on CT imaging, 5 score weightage), oral ulcer (detected on physical examination and history, 2 score weightage), and proteinuria (1.4 g/day at initial findings and sustained + 2 ~ + 3 in urine dipstick test after infectious conditions, 4 score weightage). Total score was 15 and the patient was diagnosed with SLE [3]. The patient had pleural and pericardial effusions, but had decreased effective circulating volume. Therefore, fluid therapy and antibiotics were initiated due to upper respiratory infection symptoms and signs. The serum creatinine level recovered to 3.9 mg/dL at 1 month after hospitalization, when the patient was discharged. The serum creatinine improved to 3.03 mg/dL at 2 months after first visit. Prednisolone 10 mg per day and hydroxylchloroquine sulfate 400 mg per day were started 3 months after first visit.

She was followed up for 16 months at the outpatient department and hemoglobin level was stable during first 16 months using erythropoiesis stimulating agent (ESA) and oral iron supplementation. However, at 17 months after the first visit, the serum creatinine level steadily increased and the anemia was refractory to the erythropoiesis-stimulating agent and iron supplementation treatment. At the outpatient department, the laboratory findings revealed blood urea nitrogen, serum creatinine, and hemoglobin levels of 103 mg/dL, 6.1 mg/dL, and 9.0 g/dL, respectively. She was started on HD through a tunneled cuff catheter and underwent surgery for autologous arteriovenous fistula creation. Hemoglobin level was relatively stable after initiation of HD. We thought that the cause of anemia at the initiation of HD was uremia.

**Fig. 1:**
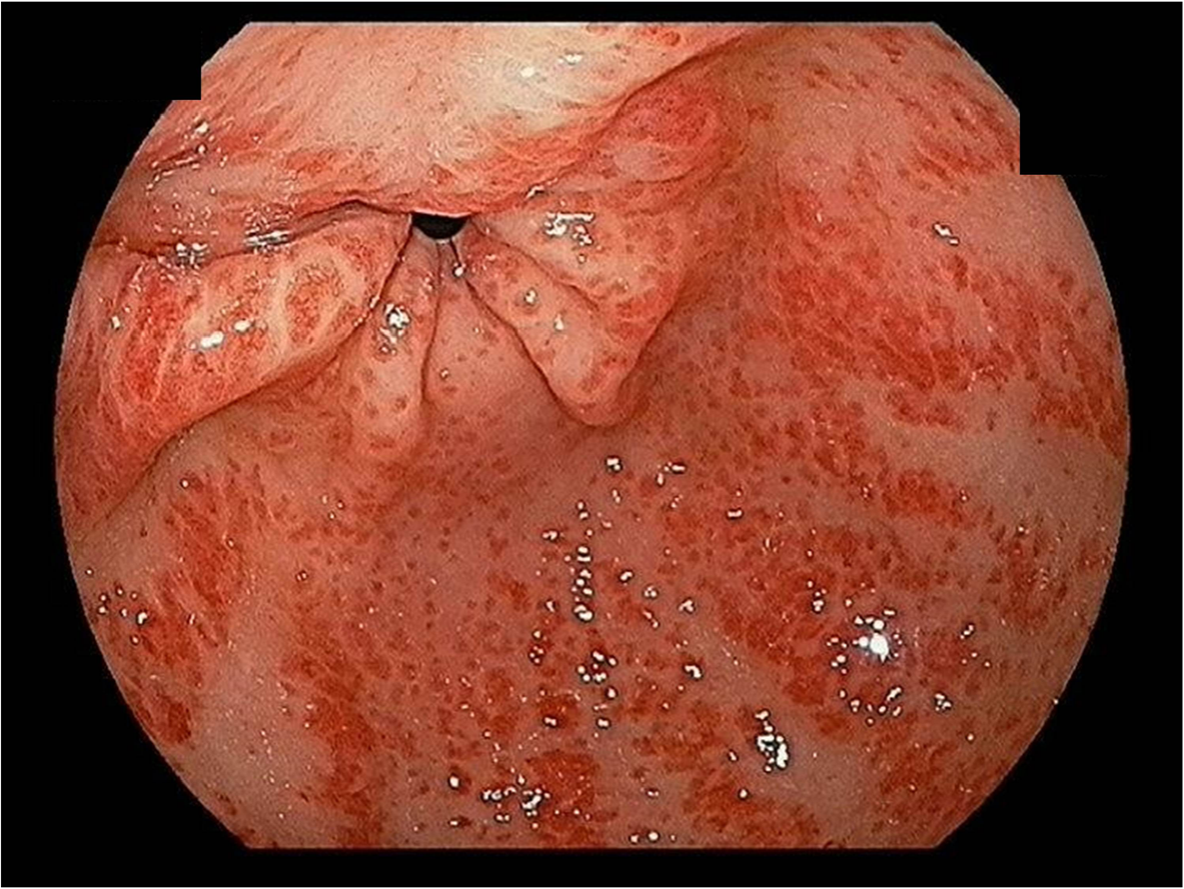
Gastrointestinal endoscopy revealed multiple erythematous and hyperemic mucosal lesions at the distal antrum without active bleeding, a finding compatible with gastric antral vascular ectasia

After 5 months from initiation of HD, hemoglobin level suddenly dropped to 5.5 g/dL and the patient complaint of dizziness and did not have change in stool nature. The anemia was evaluated using the iron status, peripheral blood (PB) smear, and GFS. Iron, transferrin saturation, and ferritin levels were 46 μg/dL, 18.5%, and 448.5 ng/mL (reference level: 5–205 ng/mL), respectively. The PB smear revealed a normocytic normochromic anemia. GFS revealed multiple erythematous raised hyperemic mucosal lesions at the distal antrum without active bleeding (Fig. 1), a finding compatible with GAVE. Anti-ulcer treatment was started using a proton pump inhibitor. Although the patient was diagnosed with GAVE, the patient did not have active bleeding focus and melena. We performed bone marrow biopsy because the definite cause of anemia was not identified. The bone marrow biopsy showed a normocellular marrow with normal iron status. Hemoglobin level was stable after transfusion and anemia did not recur.

At 9 months after initiation of HD, the patient firstly complaint of melena and emergency GFS was performed. The GFS revealed active bleeding lesion at GAVE. Bleeding focus was treated using argon plasma coagulation (APC). The hemoglobin level was stable after the treatment of GAVE. However, after first diagnosis of bleeding at GAVE, she frequently complained of symptoms or signs associated with anemia, such as dizziness, dyspnea, hypotension, melena, and hematemesis. During 18 months after the diagnosis of GAVE, she underwent four pre-emptive APC treatments for the GAVE lesions expected to bleed and four APC treatments for actively bleeding lesions. Although she frequently complained of symptoms or signs associated with anemia and had frequent GFSs with or without APC, her clinical status is relatively stable, and she is undergoing maintenance HD without anticoagulants.

## Discussion and conclusion

We wanted to present a case with multiple risk factors for GAVE, such as advanced chronic kidney disease (CKD) and SLE, and importance of repeated GFS in patients with multiple risk factors. Our case reveal that GAVE developed between first visit and 5 months after initiation of HD. Although anemia was presented at the initiation of HD, anemia was corrected after initiation of HD. We diagnosed anemia caused by uremia and did not perform repeated GFS. We definitely diagnosed GAVE at 5 months after initiation of HD. However, at first visit, GFS at local medical center may be misdiagnosed to erosive gastritis, which can be difficult to differentiate from GAVE in some cases [4, 5]. At 5 months after initiation of HD, hemoglobin level suddenly decreased, but bleeding from GAVE and melena were not observed. Therefore, we performed bone marrow biopsy. The cause of anemia at this point may be occult bleeding from GAVE, but the patient’s hemoglobin level was normalized after transfusion and stable hemoglobin level was maintained using proper ESA and iron therapy. Significant first bleeding in GAVE developed at 9 months after the initiation of HD. These findings reveal that GAVE can be misdiagnosed with diseases such as erosive gastritis and repeated GFS should be considered in patients with anemia, multiple risk factors of GAVE, and refractory to resistant to ESAs and iron, although recent performed GFS did not show GAVE.

GAVE was first reported by Rider et al., characterized with longitudinal streaks of erythematous mucosa, termed as watermelon stomach based on the striped appearance [6]. Previous studies suggested that GAVE is an acquired disease that can be caused by mechanical stress, such as portal hypertension, hormones, such as gastrin, CKD, and connective tissue disease [7].

CKD is one of the commonest health problems [1]. Patients with CKD are prone to anemia due to many factors, and gastrointestinal bleeding should be excluded. GAVE is a rare cause of anemia in these patients. A previous report suggested that the relationship between GAVE and CKD is associated with abnormalities of the gastric emptying and antral motility, vasoactive mediators, and increased gastrin levels [8]. The treatment of GAVE in patients with CKD is same as that in patients without CKD. Repeated endoscopic coagulation is important regardless of the etiology. In previous reports, patients were treated using various methods, such as APC, laser coagulation, gastrectomy, or electrocoagulation. GAVE in patients with CKD was most commonly reported in the Japanese elderly population [9, 10].

Autoimmune disorders are co-present in 60% of the patients with GAVE [2]. Many of these patients have autoantibodies. Although the association between the autoantibodies and GAVE as a pathogenesis of the disease is obscure, some investigators showed cross-reactivity of the autoantibodies with the gastric vessels [11, 12]. In our case, she had autoantibodies and CKD as risk factors of GAVE. She did not respond to ESA and iron supplementation treatment. Repeated APCs were needed to control the anemia and/or bleeding. GAVE in patients with lupus undergoing HD is even more rare. Jinga et al. reported a case of GAVE in a patient on peritoneal dialysis who had lupus [13]. To the best of our knowledge, this is the first report in Korea population.

No renal pathologic finding is an important limitation in our study. Renal biopsy should be considered for definite diagnosis of lupus nephritis, but we thought that renal pathologic finding would be not essential for future treatment plan or patient prognosis, considering risks of renal biopsy or use of strong immunosuppressants due to old age and poor general conditions. In addition, the patient refused renal biopsy. The patient can be diagnosed with renal disease by either immune or non-immune mediated injuries. The patient had normal complement level and negative result for anti-dsDNA level. We suggest that the patient had acute kidney injury on CKD regarding clinical course and laboratory findings. CKD in this case could be associated with non-immunologic injuries alone, immunologic injuries regardless of lupus, or non-immunologic injuries comined with non-active lupus nephritis rather than active lupus nephritis per se [14, 15]. Acute kidney injury may be associated with non-immunologic problems due to improvement by conservative treatment alone. In our case, immunosuppressants were started 3 months after first visit, but the serum creatinine improved from 8.87 mg/dL at first visit to 3.03 mg/dL at 2 months after first visit.

Two factors in HD patients can cause difficulty in diagnosis and treatment of gastrointestinal bleeding, such as GAVE. First, CKD is well-known as a category of anemia of chronic disease with high ferritin and low transferrin saturation levels [16]. Iron deficiency status as a manifestation of bleeding can be hidden due to underlying high ferritin and low transferrin saturation levels. The patient had high serum ferritin level from first visit to first bleeding. The relatively high ferritin level at first bleeding would be associated with combination of anemia of chronic disease and bleeding. However, serum ferritin level decreased to 71.34 ng/mL at 2nd bleeding from GAVE, inspite of oral iron therapy. C-reactive protein level was high at first visit (13.615 mg/dL), the level was normal at 1 month after initial first visit (0.19 mg/dL) and the C-reactive protein level was stable throughout the clinical course. These findings reveal that high ferritin and low transferrin saturation levels were associated with anemia of chronic disease by CKD (not for acute inflammatory status). Second, most HD patients are prone to anemia and require ESAs and iron supplementation [16]. Oral iron supplementation is associated with dark stool, which lead to difficulty in identifying grossly melena and false positive for stool occult blood test. If bleeding from gastrointestinal lesion was insidious, hemoglobin level will decrease slowly. The clinician may first consider increase in dose of ESAs rather than evaluation of bleeding. The clinician may consider gastrointestinal bleeding as a cause of anemia in unusual high dose of ESA or sudden large decrease in hemoglobin level.

This clinical case suggests that GAVE should be considered as a cause of the anemia resistant to ESAs and iron supplementation in patients with CKD and lupus.

## Data Availability

Not applicable.

## Abbreviations

ANA: Antinuclear antibody
CKD: Chronic kidney disease
GAVE: Gastric antral vascular ectasia
GFS: Gastrointestinal endoscopies
ESA: Erythropoiesis stimulating agent
HD: Hemodialysis
PB: Peripheral blood
APC: Argon plasma coagulation
